# Open-Label Crossover Oral Bioequivalence Pharmacokinetics Comparison for a 3-Day Loading Dose Regimen and 15-Day Steady-State Administration of SUBA-Itraconazole and Conventional Itraconazole Capsules in Healthy Adults

**DOI:** 10.1128/AAC.00400-20

**Published:** 2020-07-22

**Authors:** George R. Thompson, Phoebe Lewis, Stuart Mudge, Thomas F. Patterson, Bruce P. Burnett

**Affiliations:** a UC Davis School of Medicine, Department of Internal Medicine, Division of Infectious Diseases, Sacramento, California, USA; b UC Davis School of Medicine, Department of Medical Microbiology and Immunology, Sacramento, California, USA; c Department of Medical Affairs, Mayne Pharma, Inc., Raleigh, North Carolina, USA; d Department of Medical Affairs, Mayne Pharma, LLC, Melbourne, Australia; e University of Texas Health Science Center, Division of Infectious Diseases, San Antonio, Texas, USA; f South Texas Veterans Health Care System, San Antonio, Texas, USA

**Keywords:** absorption, antifungal agents, bioequivalence, itraconazole, pharmacokinetics

## Abstract

Super bioavailability (SUBA) itraconazole (S-ITZ), which releases drug in the duodenum, and conventional itraconazole (C-ITZ), which releases drug in the stomach, were compared in two pharmacokinetic (PK) studies: a 3-day loading dose study and a 15-day steady-state administration study. These were crossover oral bioequivalence studies performed under fed conditions in healthy adult volunteers. In the loading dose study, C-ITZ (two doses of 100 mg each) and S-ITZ (two doses of 65 mg each) were administered three times daily for 3 days and once on day 4 (*n* = 15). For the steady-state administration study, C-ITZ (two doses of 100 mg each) and S-ITZ (two doses of 65 mg each) were administered twice daily for 14 days and a last dose was administered 30 min after a meal on day 15 (*n* = 16). Blood samples collected throughout both studies were analyzed for ITZ and hydroxy-ITZ (OH-ITZ) levels. Least-squares geometric means were used to compare the maximum peak concentration of drug after administration at steady state prior to administration of the subsequent dose (*C*_max_ss_), the minimum drug level after administration prior to the subsequent dose (*C*_trough_), and the area under the curve over the dosing interval (AUC_tau_) of each formulation. The ratios of itraconazole (ITZ) and OH-ITZ for S-ITZ to C-ITZ were between 107% and 118% in both studies for *C*_max_ss_, *C*_trough_, and AUC_tau_, which were within the U.S. FDA-required bioequivalence range of 80% to 125%. At the end of the steady-state administration study, 13 of 16 volunteers obtained higher mean ITZ blood *C*_trough_ levels of >1,000 ng/ml when they were administered S-ITZ (81%) than when they were administered C-ITZ (44%). The study drugs were well tolerated in both studies, with similar adverse events (AEs). All treatment-emergent AEs resolved after study completion. One volunteer receiving C-ITZ discontinued due to a treatment-unrelated AE in the steady-state administration study. No serious AEs were reported. Total, trough, and peak ITZ and OH-ITZ exposures were similar between the two formulations. Therefore, SUBA-ITZ, which has 35% less drug than C-ITZ, was bioequivalent to C-ITZ in healthy adult volunteers and exhibited a safety profile similar to that of C-ITZ.

## Abstract (precis)

Super bioavailability (SUBA) itraconazole (S-ITZ), which releases drug in the duodenum, and conventional itraconazole (C-ITZ), which releases drug in the stomach, were compared in two pharmacokinetic (PK) studies: a 3-day loading dose study and a 15-day steady-state administration study. These were crossover oral bioequivalence studies performed under fed conditions in healthy adult volunteers. In the loading dose study, C-ITZ (two doses of 100 mg each) and S-ITZ (two doses of 65 mg each) were administered three times daily for 3 days and once on day 4 (*n* = 15).

## INTRODUCTION

Itraconazole (ITZ) is an orally administered antifungal agent used for the prophylaxis and treatment of invasive fungal infections. Both itraconazole and its major metabolite, hydroxyitraconazole (OH-ITZ), possess significant antifungal activity. Conventional ITZ (C-ITZ) is commercially available as a 100-mg capsule formulation or a liquid suspension; however, intrapatient differences in bioavailability, food and acid requirements for absorption, and the poor gastrointestinal tolerability of these conventional ITZ formulations limit their use (1). In a real-world study of patient blood levels of ITZ, presumably in patients administered both capsule and oral solution formulations, Wiederhold et al. (2) found that out of almost 700 levels tested, only 56.7% were found to be above 500 ng/ml, a reference level associated with few breakthrough infections in neutropenic patients (3).

A new formulation, super bioavailability (SUBA) ITZ (S-ITZ; 65 mg), has been developed (4) and approved by the U.S. FDA and is indicated for the treatment of blastomycosis, histoplasmosis, and aspergillosis (5). This novel formulation contains a solid dispersion of ITZ in a polymeric matrix, which, combined with formulation processing, enhances both dissolution and intestinal absorption. The polymeric matrix facilitates dissolution at pH levels found in the upper gastrointestinal tract, whereas the drug presentation in the C-ITZ capsule formulation requires acidic medium for dissolution. These changes in the ITZ formulation resulted in a 50-mg S-ITZ formulation, approved in Australia as well as certain European and South American countries, that demonstrated relative bioavailability that was improved by 173% compared to that of C-ITZ capsules and a 21% decrease in intrapatient variability (4). The U.S. FDA-approved prescribing information for S-ITZ recommends that it be taken with food; however, only moderate food effects were observed, as the minimum drug levels after administration prior to the subsequent dose (*C*_trough_) in the fed and fasted states are within 10% of each other (5). When a single dose of S-ITZ was coadministered with a proton pump inhibitor after establishing its steady state, an increase in ITZ plasma exposure was observed (1). These are key attributes that may be helpful in patients with subtherapeutic ITZ serum drug concentrations due to poor absorption of C-ITZ capsule formulations.

The objective of these studies was to compare the rate and extent of absorption of S-ITZ and C-ITZ in healthy adult volunteers in a crossover design and to assess the safety and tolerability of both S-ITZ and C-ITZ capsules. Two different bioequivalence studies were performed: a 3-day loading dose study and a 15-day steady-state administration study. These studies were undertaken as part of the approval process for S-ITZ required by the U.S. FDA (6).

(Data from this study were presented in part at the 29th European Congress of Clinical Microbiology & Infectious Diseases [ECCMID], Amsterdam, Netherlands, 13 to 16 April 2019.)

## RESULTS

### Study participants.

Demographic information for the volunteers in the 3-day loading dose and 15-day steady-state pharmacokinetic (PK) studies is shown in Table 1. For the 3-day loading dose PK analysis, 16 volunteers between the ages of 18 and 65 years were initially enrolled. All volunteers were administered C-ITZ and completed the C-ITZ phase, but only 15 completed S-ITZ administration and data collection, as 1 subject was removed due to a protocol deviation because the subject took a nonsteroidal anti-inflammatory drug (an excluded drug) during treatment with C-ITZ. Therefore, only data for these 15 volunteers were included in the PK and statistical analyses (Table 1). For the 15-day steady-state PK comparison, 24 different volunteers with similar racial and age distributions to those of the volunteers in the 3-day loading dose study were initially enrolled, and 16 of these volunteers completed the study. Twenty volunteers received at least one administration of S-ITZ, and 22 volunteers received at least one administration of C-ITZ. Eight volunteers discontinued participation in the study prior to the final PK sampling (day 15) for the S-ITZ phase. As such, a total of 16 volunteers were included in the PK and statistical analyses (Table 1).

**TABLE 1:** Demographic information for volunteers in both the 3-day loading dose and 15-day steady-state administration studies^*a*^

Demographic characteristic	3-day loading dose study		15-day steady-state administration study	
	Volunteers enrolled (*n* = 16)	PK statistical data sets (*n* = 15)	Volunteers enrolled (*n* = 24)	PK statistical data sets (*n* = 16)
Mean ± SD age (yr)	40 ± 11	41 ± 11	34 ± 10	33 ± 9
No. (%) of volunteers in the following age group (yr):				
<18	0 (0)	0 (0)	0 (0)	0 (0)
18–40	7 (43.8)	6 (40.0)	18 (75.0)	12 (75.0)
41–65	9 (56.3)	9 (60.0)	6 (25.0)	4 (25.0)
65–75	0 (0)	0 (0)	0 (0)	0 (0)
>75	0 (0)	0 (0)	0 (0)	0 (0)
Mean ± SD BMI (kg/m^2^)	26.4 ± 3.6	26.5 ± 3.7	27.7 ± 2.6	28.1 ± 2.5
Mean ± SD wt (kg)	76.9 ± 14.1	76.8 ± 14.5	81.3 ± 11.6	82.3 ± 12.5
Mean ± SD ht (cm)	170.3 ± 9.8	169.8 ± 10.0	171.2 ± 9.1	170.7 ± 8.9
No. (%) of volunteers by gender				
Female	9 (56.3)	8 (53.3)	11 (45.8)	7 (43.8)
Male	7 (43.8)	7 (46.7)	13 (54.2)	9 (56.3)
No. (%) of volunteers by race or ethnicity				
Asian	1 (6.3)	1 (6.7)	1 (4.2)	0 (0)
Black or African American	13 (81.3)	12 (80.0)	18 (75.0)	12 (75.0)
White	2 (12.5)	2 (13.3)	1 (4.2)	1 (6.3)
Hispanic or Latino	0 (0)	0 (0)	0 (0)	0 (0)
Multiracial			2 (8.3)	2 (12.5)
American Indian or Alaska Native			2 (8.3)	1 (6.3)

a BMI, body mass index; *n*, number of subjects; PK, pharmacokinetics.

### Pharmacokinetics.

Arithmetic mean ITZ predose plasma concentrations (*C*_pd_) for both treatments increased steadily on days 1 through 4 in the loading dose study. On days 2, 3, and 4, the arithmetic mean and standard deviation (SD) ITZ *C*_pd_ for S-ITZ were 506.8 ± 125.8 ng/ml, 760.1 ± 191.7 ng/ml, and 1,029.4 ± 255.9 ng/ml, respectively, which were ∼22% to 29% higher than the values of 394.1 ± 106.8 ng/ml, 599.0 ± 201.1 ng/ml, and 845.4 ± 226.4 ng/ml, respectively, for C-ITZ. The results of the PK and statistical analyses for ITZ and OH-ITZ for the 3-day loading dose study are shown in Table 2. Administration of S-ITZ resulted in a nonsignificant ∼10% higher geometric mean area under the curve ITZ for over the dosing interval (AUC_tau_) for S-ITZ compared to C-ITZ. The geometric mean *C*_trough_ following S-ITZ administration was 7% higher than that following C-ITZ administration. The geometric mean maximum peak concentration of drug after administration at steady state prior to administration of the subsequent dose (*C*_max_ss_) for S-ITZ was 1,055 ng/ml ITZ after breakfast on day 4, which was 14% higher than the ITZ level of 921.3 ng/ml reached with conventional ITZ. The PK parameters for OH-ITZ measured from both formulations followed a pattern in terms of bioavailability similar to that for the ITZ plasma blood levels for the S-ITZ/C-ITZ ratios for *C*_max_ss_ (109.6%), *C*_trough_ (108.8%), and AUC_tau_ (111.6%) (Table 2). All PK parameters were well within the 90% confidence interval (CI).

**TABLE 2:** Pharmacokinetic and statistical analyses of itraconazole and hydroxyitraconazole for the 3-day loading dose PK comparison^*a*^

Parameter	Results for ITZ				Results for OH-ITZ			
	Geometric mean value		S-ITZ/C-ITZ ratio (%)	90% CI	Geometric mean value		S-ITZ/C-ITZ ratio (%)	90% CI
	S-ITZ	C-ITZ			S-ITZ	C-ITZ		
*C*_max_ss_ (ng/ml)	1,055.3	921.3	114.54	103.40–126.88	1,691.0	1,543.3	109.57	101.45–118.35
*C*_trough_ (ng/ml)	881.1	820.6	107.37	95.58–120.62	1,640.8	1,508.8	108.75	100.39–117.80
AUC_tau_ (ng · h/ml)	6,881.7	6,236.2	110.35	100.10–121.66	12,632.7	11,317.0	111.63	103.15–120.80

a AUC_tau_, area under the curve over the dosing interval; CI, confidence interval; C-ITZ, conventional itraconazole; *C*_max_ss_, maximum peak concentration of drug after administration at steady state prior to administration of the subsequent dose; *C*_trough_, minimum drug level after administration prior to the subsequent dose; S-ITZ, SUBA-itraconazole.

**FIG 1:**
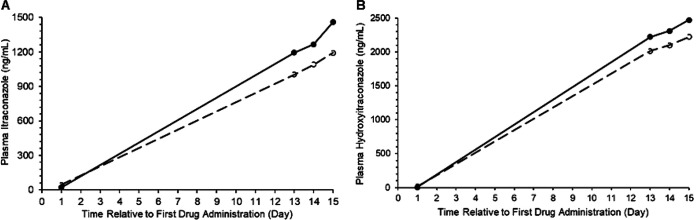
Day 1 to day 15 plasma levels of itraconazole (A) and hydroxyitraconazole (B) for the SUBA-itraconazole (solid line) and conventional itraconazole (dashed line) formulations.

In the 15-day study, ITZ predose blood levels for both treatments increased steadily from the first day of administration to days 13, 14, and 15. The arithmetic means and standard deviations for *C*_pd_ for S-ITZ were 21.5 ± 32.5 ng/ml, 1,194.3 ± 288.8 ng/ml, 1,266.5 ± 338.9 ng/ml, and 1458.6 ± 402.0 ng/ml on days 1, 13, 14, and 15, respectively, whereas those for C-ITZ were 43.8 ± 66.1 ng/ml, 1,003.1 ± 232.9 ng/ml, 1,090.3 ± 269.8 ng/ml, and 1,192.8 ± 286.1 ng/ml, respectively. Consistent with the findings in the loading dose study, predose ITZ blood levels on days 13, 14, and 15 in the steady-state PK study were between 16% and 22% higher for the S-ITZ formulation than for the C-ITZ formulation. The results of the PK and statistical analyses for ITZ and OH-ITZ for the 15-day steady-state administration study are shown in Table 3. At steady state, 65-mg S-ITZ capsule administration resulted in greater absorption than 100-mg C-ITZ capsule administration, with ITZ showing an ∼12% higher *C*_max_ss_, an 18% greater *C*_trough_, and an 11% better AUC_tau_ with S-ITZ than with C-ITZ. These values were still within the acceptable confidence intervals, thus demonstrating bioequivalence. The measured PK parameters for OH-ITZ at steady state from both formulations followed a similar pattern in terms of bioavailability. The values of *C*_max_ss_, *C*_trough_, and AUC_tau_ for itraconazole after administration of the 65-mg S-ITZ formulation were higher than those after administration of the 100-mg C-ITZ formulation (Table 3). The values of all PK parameters were also within the 90% confidence intervals. No significant differences between formulations were found for any PK parameters for ITZ or OH-ITZ at steady state. The median time to *C*_max_ss_ (*T*_max_ss_) for S-ITZ was 7 h, whereas it was 5 h for conventional ITZ, consistent with dissolution and absorption at a lower point in the gastrointestinal tract. Mean ITZ and OH-ITZ plasma levels after S-ITZ administration were higher than those after conventional ITZ administration on days 13 to 15 (Fig. 1A and B).

**TABLE 3:** Pharmacokinetic and statistical analysis of itraconazole and hydroxyitraconazole for the 15-day steady-state PK comparison^*a*^

Parameter	Results for ITZ				Results for OH-ITZ			
	Geometric mean value		S-ITZ/C-ITZ ratio (%)	90% CI	Geometric mean value		S-ITZ/C-ITZ ratio (%)	90% CI
	S-ITZ	C-ITZ			S-ITZ	C-ITZ		
*C*_max_ss_ (ng/ml)	1,632.2	1,457.5	111.99	104.87–119.59	2,613.6	2,338.9	111.75	105.89–117.93
*C*_trough_ (ng/ml)	1,187.4	1,004.9	118.16	110.20–126.69	2,335	2,061.7	113.26	107.39–119.44
AUC_tau_ (ng · h/ml)	15,562.1	14,065.1	110.64	104.01–117.70	28,143.4	25,488.1	110.42	104.28–116.91

a AUC, area under the curve over the dosing interval; CI, confidence interval; C-ITZ, conventional itraconazole; *C*_max_ss_, maximum peak concentration of drug after administration at steady state prior to the subsequent dose; *C*_trough_, minimum drug level after administration prior to the subsequent dose; S-ITZ, SUBA-itraconazole.

**FIG 2:**
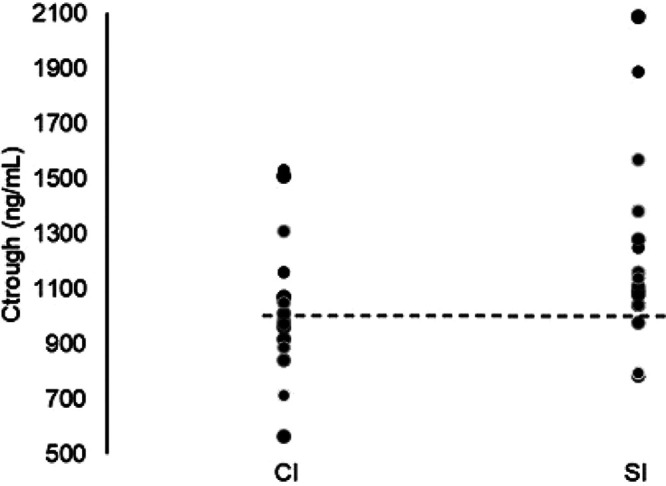
Fifteen-day steady-state patient-level data for the geometric mean *C*_trough_ values for conventional itraconazole (CI) and SUBA-itraconazole (SI). The dotted line represents the geometric mean of the conventional itraconazole plasma trough level (1,034 ng/ml).

When the ratio of steady-state *C*_trough_ ITZ plasma levels from S-ITZ to C-ITZ was calculated (1.18) and then corrected for the dosage of each formulation [e.g., 1.18 multiplied by (100 mg/65 mg)], the relative bioavailability of S-ITZ was 1.82 times that of conventional ITZ. From the volunteer data, 81% (13/16) of volunteers receiving S-ITZ and 44% (7/16) of volunteers receiving conventional ITZ achieved ITZ *C*_trough_ values of 1,034 ng/ml at steady state, which was the average *C*_trough_ value for the C-ITZ group (Fig. 2).

**FIG 3:**
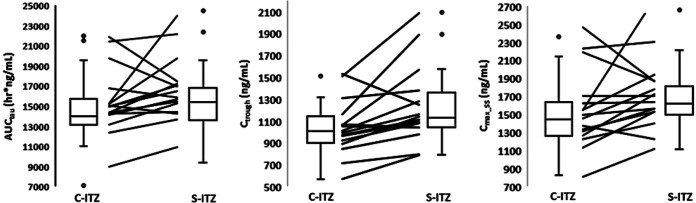
Fifteen-day steady-state patient-level crossover PK data for the geometric mean plasma AUC_tau_ (left), *C*_trough_ (middle), and *C*_max_ss_ (right) values for itraconazole achieved with the conventional itraconazole (C-ITZ) and SUBA-itraconazole (S-ITZ) formulations. Data are represented as whisker plots for both each group and the individual volunteer response (lines).

**FIG 4:**
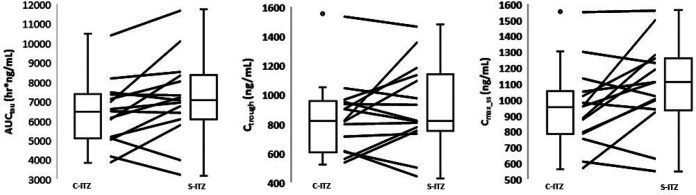
Three-day steady-state patient-level crossover PK data for the geometric mean plasma AUC_tau_ (left), *C*_trough_ (middle), and *C*_max_ss_ (right) values for the conventional itraconazole (C-ITZ) and SUBA-itraconazole (S-ITZ) formulations. Data are represented as whisker plots for both each group and the individual volunteer response (lines).

Volunteers crossed over from one period of administration of either C-ITZ or S-ITZ to a second period with dosing with the opposite drug. Twelve of 16 and 13 of 16 healthy volunteers administered S-ITZ attained higher AUC_tau_ and *C*_max_ss_ and *C*_trough_ values, respectively, for ITZ than the same volunteers administered C-ITZ in the 15-day steady-state administration study (Fig. 3). Whisker plots showed that higher median and quartile ITZ levels for all three PK parameters were achieved when volunteers were administered S-ITZ than when volunteers were administered C-ITZ. Volunteer-level data from the 3-day loading dose PK study were mixed. In terms of overall exposure and peak plasma levels on day 4, three volunteers had higher peak plasma levels of ITZ when they were administered C-ITZ than when they were administered S-ITZ, and two had approximately the same levels (Fig. 4, left and right panels). In terms of the ITZ *C*_trough_ in plasma, out of 15 healthy volunteers, 6 volunteers receiving C-ITZ had higher levels than volunteers receiving S-ITZ and 3 had levels that were approximately the same as those of volunteers receiving S-ITZ (Fig. 4, middle panel). Similar to the findings of the 15-day steady-state administration study, whisker plots clearly demonstrated that higher median and quartile ITZ levels for all three PK parameters were achieved after 3 days when volunteers were administered S-ITZ than when they were administered C-ITZ. The percent covariances for both S-ITZ and C-ITZ [(arithmetic mean/standard deviation) × 100] for the 3-day and 15-day studies were similar for all PK parameters.

### Safety and tolerability.

The administration of the study drugs was generally well tolerated by the volunteers participating in both studies. In the 3-day loading dose study, there were 10 reports of at least one treatment-emergent adverse event (TEAE) in 4 volunteers (3 in the S-ITZ group versus 7 in the C-ITZ group), of which only 1 was deemed to have been related to the administered drug (headache after receipt of C-ITZ) (Table 4). No volunteer discontinued from the study due to a TEAE. All reported TEAEs were mild in severity and resolved prior to the end of the study. No severe adverse events (SAEs) were reported during the conduct of this study, and none of the TEAEs had a significant impact on the safety of the volunteers or on the integrity of the study results.

**TABLE 4:** Treatment-emergent adverse events in the 3-day loading dose study^*a*^

System organ class term	No. (%) of volunteers with TEAEs		
	S-ITZ (*n* = 3)	C-ITZ (*n* = 7)	Total (*n* = 10)
Gastrointestinal disorders	2 (66.7)	1 (14.3)	3 (30.0)
Abdominal pain	1 (33.3)	0 (0)	1 (10.0)
Nausea	1 (33.3)	0 (0)	1 (10.0)
Toothache	0 (0)	1 (14.3)	1 (10.0)
General disorders and administration site condition	0 (0)	1 (14.3)	1 (10.0)
Pyrexia	0 (0)	1 (14.3)	1 (10.0)
Investigations	1 (33.3)	0 (0)	1 (10.0)
Platelet count decreased	1 (33.3)	0 (0)	1 (10.0)
Nervous system disorders	0 (0)	1 (14.3)	1 (10.0)
Headache	0 (0)	1 (14.3)	1 (10.0)
Reproductive system and breast disorders	0 (0)	2 (28.6)	2 (20.0)
Dysmenorrhea	0 (0)	2 (28.6)	2 (20.0)
Respiratory, thoracic, and mediastinal disorders	0 (0)	2 (28.6)	2 (20.0)
Epistaxis	0 (0)	2 (28.6)	2 (20.0)

a C-ITZ, conventional itraconazole; S-ITZ, SUBA-itraconazole; TEAEs, treatment-emergent adverse events.

In the 15-day steady-state administration study, there were 25 reports of at least one TEAE in 11 volunteers, and of these, 8 TEAEs in 5 volunteers were deemed to have been related to the administered drug (a possible or probable relationship to the study drug) (Table 5). Nine volunteers receiving S-ITZ reported 16 potential TEAEs, and 4 volunteers receiving C-ITZ reported 9 potential TEAEs. One volunteer administered C-ITZ withdrew from the study due to a treatment-unrelated TEAE (a toothache), which was mild in severity and which resolved. All TEAEs were mild in severity and resolved prior to the end of the study. No SAEs were reported during the conduct of this study, and none of the TEAEs had a significant impact on the safety of the volunteers or on the integrity of the 15-day steady-state administration study results.

**TABLE 5:** Treatment-emergent adverse events in the 15-day steady-state administration study^*a*^

System organ class term	No. (%) of volunteers with TEAEs		
	S-ITZ (*n* = 16)	C-ITZ (*n* = 9)	Total (*n* = 25)
Gastrointestinal disorders	9 (56.3)	1 (11.1)	10 (40.0)
Abdominal pain	1 (6.3)	0 (0)	1 (4.0)
Abdominal pain, upper	1 (6.3)	1 (11.1)	2 (8.0)
Abdominal tenderness	1 (6.3)	0 (0)	1 (4.0)
Constipation	2 (12.5)	0 (0)	2 (8.0)
Nausea	3 (18.8)	0 (0)	3 (12.0)
Toothache	1 (6/3)	0 (0)	1 (4.0)
Injury, poisoning, and procedural complications	1 (6.3)	0 (0)	1 (4.0)
Arthropod bite	1 (6.3)	0 (0)	1 (4.0)
Investigations	0 (0)	3 (33.3)	3 (12.0)
Alanine aminotransferase level increased	0 (0)	1 (11.1)	1 (4.0)
Neutrophil count increased	0 (0)	1 (11.1)	1 (4.0)
White blood cell count increased	0 (0)	1 (11.1)	1 (4.0)
Musculoskeletal and connective tissue disorders	1 (6.3)	2 (22.2)	3 (12.0)
Back pain	1 (6.3)	0 (0)	1 (4.0)
Neck pain	0 (0)	2 (22.2)	2 (8.0)
Nervous system disorders	5 (31.3)	1 (11.1)	6 (24.0)
Dizziness	1 (6.3)	1 (11.1)	2 (8.0)
Headache	4 (25.0)	0 (0)	3 (12.0)
Reproductive system and breast disorders	0 (0)	2 (22.2)	2 (8.0)
Dysmenorrhea	0 (0)	2 (22.2)	2 (8.0)

a C-ITZ, conventional itraconazole; S-ITZ, SUBA-itraconazole; TEAEs, treatment-emergent adverse events.

## DISCUSSION

The results of these two PK comparisons demonstrate that 65-mg S-ITZ capsules are bioequivalent to 100-mg C-ITZ capsules when administered under fed conditions after a 3-day loading dose regimen and at steady state after 15 days. The U.S. FDA requires any new formulation of a previously approved drug with a delivery mechanism different from that of the previously approved drug to be bioequivalent for absorption and to have the corresponding safety (7). Here, we demonstrate that S-ITZ capsules exhibit oral bioequivalence to C-ITZ capsules with 35% less drug and that volunteers receiving the S-ITZ formulation were more likely to reach therapeutic drug levels (>1,000 ng/ml) than volunteers receiving the C-ITZ formulation when administered at steady state.

When C-ITZ was first approved in 1992, this drug was the first mold-active oral azole and advanced the treatment of endemic and systemic fungal infections (8). Unfortunately, this formulation has a limited 55% absolute bioavailability and needs to be taken with a full meal or with an acidic beverage. Acid-suppressive drugs further reduce the absorption of the capsule formulation of C-ITZ by approximately half (9). To address these issues and increase the absorption of C-ITZ, an oral solution was developed and approved in 1997 (10). This oral solution formulation must be taken under fasted conditions but increases the absolute bioavailability to only about 72%, and many patients find the solution unpalatable. In subsequent years, other azoles have been approved, but ITZ remains the treatment of choice for blastomycosis, histoplasmosis, and chronic pulmonary aspergillosis. Though C-ITZ is orally available from the capsule and oral solution formulations and has broad-spectrum activity (11, 12), difficulty in achieving therapeutic blood levels due to food effects and acid-suppressive effects have limited the use of ITZ. One study found that ITZ was undetectable in ∼40% of blood samples tested (4). SUBA-itraconazole was developed to solve these difficulties and provides a more consistent ITZ formulation which is less affected by gastric acid-suppressive agents and which demonstrates comparable absorption under both fed and fasted conditions.

When a steady state of omeprazole was established after 6 days of administration in healthy volunteers and then two 65-mg S-ITZ capsules were administered, there was a 22% increase in the total plasma exposure (the area under the curve from time zero to infinity) and a 31% increase in the peak plasma exposure of ITZ (1). Among patients who had undergone allogeneic hematological stem cell transplant (HSCT) or autologous HSCT or who had been treated for hematological malignancies and received antifungal prophylaxis with the S-ITZ formulation that has been approved in Australia, Europe, and South America at 200 mg (four 50-mg capsules) twice daily (BID) (13), 54 of 98 patients were on acid suppression regimens (14). Itraconazole target trough levels of >500 ng/ml were reached in these patients, despite a significant reduction of 180 ng/ml at steady state compared to the level in patients receiving no acid suppression (*P* < 0.01). These results suggest that the S-ITZ formulation is far less affected by acid suppression than conventional ITZ (9).

Lindsay et al. (1) found that food reduced overall exposure (AUC_tau_) by ∼22% and peak plasma concentrations by ∼27% compared with the values achieved under fasted conditions when S-ITZ was administered. By comparison, when C-ITZ capsules were administered in the fasted state, absorption was reduced to 40% to 60% of the absorption achieved by administration in a fed state (15, 16). In a recent head-to-head single-dose comparison of S-ITZ to C-ITZ, overall exposure (AUC_tau_) was ∼124% and the peak plasma concentration was ∼162% for S-ITZ compared to the values achieved with C-ITZ under fasted conditions (17). Under fed conditions, overall exposure (AUC_tau_) and the peak plasma concentration for S-ITZ were ∼90% and ∼85% of the values achieved with conventional ITZ, respectively. These results suggest that food effects are less pronounced for the 65-mg S-ITZ capsule formulation than for the 100-mg C-ITZ capsule formulation.

Previous studies have found that plasma trough ITZ levels must be >500 ng/ml to achieve therapeutic efficacy against fungal infections for prophylaxis and preferably must be between 1,000 and 2,000 ng/ml for treatment (18–23). Plasma trough levels of ITZ below 500 ng/ml are predictive of therapeutic failure (3). The toxicity levels of plasma ITZ are difficult to determine, but studies suggest that trough levels of between 2,000 and 5,000 ng/ml have increased side effect profiles (24, 25). In the 3-day loading dose study described here, S-ITZ achieved a plasma trough level of 881 ng/ml, whereas C-ITZ achieved a plasma trough level of 821 ng/ml. The 15-day steady-state trough levels for ITZ were 1,187 and 1,001 ng/ml for S-ITZ and C-ITZ, respectively. Neither formulation achieved ITZ *C*_max_ss_ or trough levels that would suggest toxicity, based on previous reports. In addition, the 65-mg S-ITZ formulation achieved levels similar to those achieved under a fed condition by Lindsay et al. (1). The steady-state trough levels from this study obtained by administering the 65-mg S-ITZ formulation were also similar to those achieved in other studies of the 50-mg S-ITZ formulation approved in Australia, Europe, and South America (14, 26).

The adverse events observed in these studies were similar to those previously described with S-ITZ (1, 8, 14, 26). Mild gastrointestinal complaints (nausea or abdominal pain), headache, and transaminase level elevation are all well described with the use of ITZ and lead to treatment discontinuation. The PK studies described here demonstrate that the 65-mg S-ITZ formulation is bioequivalent to the 100-mg C-ITZ formulation under fed conditions for a 3-day loading dose regimen and over a 15-day steady-state time frame and that the formulations have similar side effect profiles. The greater bioavailability with less pronounced food effects of the S-ITZ formulation (1, 17) may provide greater utility than the conventional form of ITZ for the treatment of endemic and systemic mycosis in patients. A head-to-head study comparing S-ITZ (Tolsura) to C-ITZ for the treatment of endemic mycoses (ClinicalTrials.gov registration number NCT03572049) is under way to evaluate safety and efficacy differences between the formulations (27).

## MATERIALS AND METHODS

### Study participants.

Volunteers enrolled in the two studies were healthy, nonsmoking male and female individuals 18 to 55 years of age with body mass indexes (BMIs) of between ≥18.0 and ≤33.0 kg/m^2^. They had normal vital signs, electrocardiograms (ECG), and laboratory values. Childbearing-age females participating in the study were required to use acceptable, effective methods of contraception. Volunteers were excluded from the study if there was a known history or presence of clinically significant conditions or diseases or if they were on concomitant medications (prescription and/or over-the-counter medications). Institutional Review Board (IRB) approval of both study protocols was obtained from Salus IRB (Austin, TX). All volunteers gave written consent prior to any study procedure.

### Clinical study medications.

The drug products used to compare bioequivalence in these two PK clinical studies were Tolsura capsules (65 mg SUBA-ITZ; Mayne Pharma) and Sporanox capsules (100 mg ITZ; Janssen Pharmaceuticals).

### Clinical study designs.

Two PK clinical studies were performed to test for bioequivalence: a 3-day loading dose study and a 15-day steady-state administration comparison of S-ITZ capsules to C-ITZ capsules. Both studies had an open-label, multiple-dose, randomized, two-period, two-treatment, two-sequence, crossover design to evaluate the comparative bioavailability of ITZ as well as its metabolite, OH-ITZ, from S-ITZ and C-ITZ capsules after administration under fed conditions. The administration of the two formulations of ITZ was separated by 21 and 14 days for the 3-day loading dose study and the 15-day steady-state administration study, respectively, corresponding to greater than five times the expected half-life of ITZ, to prevent any possible carryover.

In the 3-day loading dose PK study, volunteers were administered two 65-mg S-ITZ capsules or two 100-mg C-ITZ capsules three times daily (TID) on days 1 to 3 after consumption of a high-fat, high-calorie meal and once on the morning of day 4 after consumption of a high-fat, high-calorie breakfast. The concentrations of ITZ and OH-ITZ in plasma were measured by high-performance liquid chromatography-tandem mass spectrometry (HPLC-MS/MS) from predose samples collected on the mornings of days 1, 2, and 3 and over an 8-h interval after dosing (1, 2, 4, 6, and 8 h) on day 4 in both periods. In the 15-day steady-state PK study, the volunteers were administered two 65-mg S-ITZ capsules or two 100-mg C-ITZ capsules twice daily (BID) on days 1 to 14 after completion of the consumption of a high-fat, high-calorie meal and once on the morning of day 15 after also consuming a high-fat, high-calorie breakfast. The concentrations of ITZ and OH-ITZ in plasma were also measured by HPLC-MS/MS from predose samples collected on the mornings of days 1, 13, 14, and 15 and over a 12-h interval after dosing (0.5, 1, 1.5, 2, 2.5, 3, 3.5, 4, 5, 6, 7, 8, 10, and 12 h) on day 15 in both periods. Adverse events were monitored in both studies to assess safety.

### Safety and tolerability.

All volunteers who received at least one dose of S-ITZ or C-ITZ in either study were evaluated for the frequency and severity of AEs to determine the safety of the formulations. All TEAEs were classified according to the current version of the *Medical Dictionary for Regulatory Activities* (version 20.0) (28) and reported with respect to severity, duration, relationship to study drugs, and action taken. Vital signs measurements (blood pressure [BP], pulse rate [PR], respiration rate [RR], and temperature), electrocardiogram (ECG) recordings, safety clinical laboratory tests (liver and kidney function tests), and a physical examination were performed.

### Statistical analysis.

Pharmacokinetic parameters were estimated using a noncompartmental approach on days 1 to 3 (*C*_pd_) and day 4 (AUC_tau_, *C*_max_ss_, *T*_max_ss_, *C*_trough_, *C*_pd_) for the loading dose study and on day 15 (AUC_tau_, *C*_max_ss_, *T*_max_ss_, *C*_trough_, *C*_pd_) for the steady-state administration study. Data for volunteers for whom estimation of the *C*_max_ss_, *C*_trough_, and AUC_tau_ parameters on days 4 and 15 for the loading dose and steady-state studies, respectively, was possible and who complied with all protocol requirements were included in the PK and statistical analyses.

Descriptive statistics for the PK parameters for ITZ and OH-ITZ were calculated by treatment for volunteers included in the PK data sets. Descriptive statistics for *C*_pd_ were calculated for ITZ and OH-ITZ by study day and treatment. Descriptive statistics included the number of observations, arithmetic mean, standard deviation (SD), geometric mean (where applicable), coefficient of variation (CV), median, minimum, and maximum and were calculated using the PROC GLM procedure from SAS software (version 9.4). Analysis of variance (ANOVA) was performed on the log-transformed values of the *C*_max_ss_, *C*_trough_, and AUC_tau_ parameters. The significance of the sequence, period, treatment, and volunteer (sequence) effects was tested. Using the same statistical model, the least-squares means, the differences between the treatment least-squares means, and the corresponding standard errors of these differences were estimated for the log-transformed values of the *C*_max_ss_, *C*_trough_ and AUC_tau_ parameters.

The day 13, 14, and 15 predose ITZ levels were evaluated to assess the achievement of steady prior to PK sampling day on day 15 for both formulations. Helmert contrasts were obtained for day 13 versus the average of days 14 and 15, and for day 14 versus day 15 mean predose concentrations of ITZ. Chow & Liu (29) describe a test of nonsignificance whereby sequential linear contrasts are produced.

The first contrast tested compares the mean concentration at the first time point to the pooled mean over all remaining time points. The second contrast compares the mean at the second time point to the pooled mean over all remaining time points. Testing continues until the contrast is not statistically significant. The Helmert contrast for the day 14 versus day 15 comparison is statistically significant (α = 0.05) for the combined analysis (*P* = 0.0011), indicating that steady state was not reached by day 15. Nonetheless, the approach described by Chow and Liu (29) may not be favorable for demonstrating the achievement of steady state with larger sample sizes or smaller variations in predose levels across days. A computation of the 95% simultaneous CIs for the day 13 versus the average of days 14 and 15 contrast and for the day 14 versus day 15 contrast is proposed as a variant to the approach described by Chow and Liu (29). Employing standard bioequivalence limits of 80.00 to 125.00%, it can be concluded that steady state was achieved, as the 95% CIs for both contrasts are within 80.00 to 125.00%.
